# A case to hear: Ears hard as rock

**DOI:** 10.1016/j.jdcr.2024.10.028

**Published:** 2025-01-28

**Authors:** Lynn Midani, Kimberly Artounian, Sally Midani, Nikifor K. Konstantinov

**Affiliations:** a Department of Dermatology, University of New Mexico, Albuquerque, New Mexico; b Department of Radiology, University of New Mexico, Albuquerque, New Mexico; c Department of Dermatology & Internal Medicine, University of New Mexico, Albuquerque, New Mexico

**Keywords:** Addison's disease, autoimmune polyendocrine syndrome type 2, petrified ears

## History

**Fig 1:**
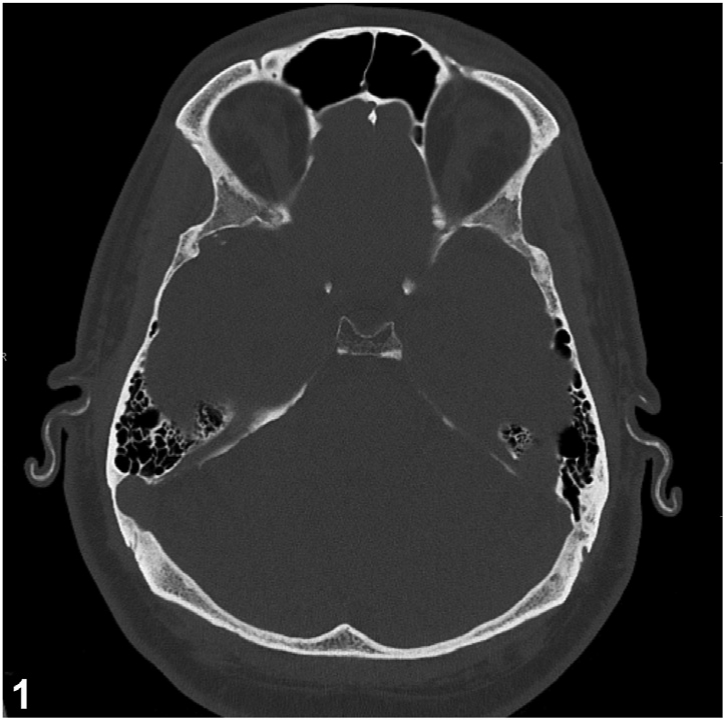

A 48-year-old male with a long-standing history of Addison's disease, Hashimoto's thyroiditis, and immunoglobulin A (IgA) vasculitis presented to the clinic for progressive stiffening of his ears. He noted a 10-year history of nonpainful stiffening of both ears, without acute hearing loss or tinnitus. On exam, his bilateral ear helices and antihelices were “rock-hard” to palpation (Supplementary Video 1, available via Mendeley at https://data.mendeley.com/datasets/vzbs84rj2p/1). The earlobes were spared and there was no evidence of inflammation. Computed tomography imaging of the ears demonstrated diffuse, smooth ossification of the auricles bilaterally (Fig 1).

**Question 1: What is the most likely diagnosis?**

A. Relapsing polychondritis
B. Cauliflower ear deformity
C. Tophaceous Gout
D. Petrified ears
E. IgA vasculitis

**Answers:**

A. Relapsing polychondritis – Incorrect. Relapsing polychondritis is a rare autoimmune condition characterized by recurrent inflammation of the auricular, articular, nasal, and tracheal cartilage. Auricular involvement is present in 90% of cases and often presents with discoloration and pain, sparing the ear lobes.^1^ Our patient’s lack of ear inflammation, symptoms, and imaging findings are not consistent with this diagnosis.
B. Cauliflower ear deformity – Incorrect. Cauliflower ear deformity is due to hematoma formation within the outer ear after mechanical trauma. If not evacuated, the hematoma can disrupt the blood supply to the auricular cartilage, leading to necrosis, inflammation, and fibrocartilaginous overgrowth. Ear examination will show contour irregularity and swelling. A history will often reveal recent blunt trauma.
C. Tophaceous Gout – Incorrect. Tophaceous gout results in tophi-tissue deposits of monosodium urate crystals associated with granulomatous inflammation. They present as hard, chalky, yellow-white papules and nodules, commonly on the joints of the hands and feet but can present on the helical rim and antihelix of the ears.^2^ Risk factors include age, male sex, postmenopausal status, immunosuppressive agents, and myeloproliferative disease.
D. Petrified ears – Correct. Petrified ears are a clinical entity which describe auricular cartilage hardening due to ectopic calcification or ossification. Many causes have been reported, including frostbite, local trauma, inflammation, and endocrine disorders, particularly adrenal insufficiency.^3^
E. IgA vasculitis – Incorrect. IgA vasculitis does not manifest with ear stiffening and auricular ossification. Cutaneous features of IgA vasculitis include palpable purpura and rarer findings may also include periorbital edema.

**Question 2: This patient’s condition is associated with which of the following?**

A. Autoimmune polyendocrine syndrome type 2
B. Nasal chondritis
C. Cutaneous vasculitis (may be seen with relapsing polychondritis)
D. Positive antiproteinase 3 (anti-PR3)
E. Myelodysplastic syndrome

**Answers:**

A. Autoimmune polyendocrine syndrome type 2 – Correct. Calcification of articular cartilage, or petrified ears is associated with endocrinopathies such as autoimmune polyendocrine syndrome type 2 and adrenal insufficiency. Autoimmune polyendocrine syndrome type 2 is characterized by at least 2 of the 3 following endocrinopathies; type one diabetes mellitus, autoimmune thyroiditis, and Addison’s disease. Endocrinopathies have been reported to cause ectopic calcium deposition but the mechanism is unknown. There is no standardized protocol for assessing patients with petrified ears, however evaluation may include assessing for underlying thyroid, adrenal, and pituitary disorders.^4^ Our patient’s lab work, including parathyroid hormone, phosphorus, glutamic acid decarboxylase, and procollagen type 1, to rule out other causes of ossification, were unremarkable.
B. Nasal chondritis – Incorrect. Nasal chondritis is a common sign of relapsing polychondritis. There is no association of petrified ears with nasal chondritis.
C. Cutaneous vasculitis (may be seen with relapsing polychondritis) – Incorrect. Petrified ears are not associated with cutaneous vasculitis.
D. Positive antiproteinase 3 (anti-PR3) – Incorrect. Antiproteinase 3 antibodies are associated with granulomatosis with polyangiitis, a necrotizing vasculitis affecting small to medium-sized blood vessels.
E. Myelodysplastic syndrome – Incorrect. Myelodysplastic syndrome is associated with relapsing polychondritis. In patients with hematologic abnormalities and relapsing polychondritis, VEXAS syndrome (vacuoles, E1 enzyme, X-linked, autoinflammatory, somatic) should be considered.

**Question 3: Which of the following statements regarding this diagnosis is correct?**

A. Females are more likely to experience this condition than males
B. Computed tomography is the gold standard diagnostic modality
C. Treatment of this condition requires immunosuppressive medications
D. This condition may present years before the onset of underlying systemic disease
E. Patients are more likely to present with unilateral involvement

**Answers:**

A. Females are more likely to experience this condition than males – Incorrect. The incidence rate is higher in males.
B. Computed tomography is the gold standard diagnostic modality – Incorrect. There is no gold standard for diagnosis, however skull X-ray and computed tomography of the temporal bone can aid in diagnosis and demonstrate dense opacities within the cartilage. Biopsy for histopathologic examination is not required, but can aid in distinguishing between auricular calcification and ossification. In our case, a biopsy was considered but ultimately not performed, as it would not have affected our patient’s management.
C. Treatment of this condition requires immunosuppressive medications – Incorrect. There is no known treatment to reverse the calcification or ossification, including treatment of underlying metabolic abnormalities. Symptomatic patients with discomfort may choose to undergo surgery. Wedge resection of the affected cartilage or conchal reduction surgery has been reported to show improvement in patient discomfort. Our patient was asymptomatic and did not undergo treatment. He was referred to endocrinology for management of his endocrinopathies.
D. This condition may present years before the onset of underlying systemic disease – Correct. This condition may present years before the onset of underlying systemic disease such as Addison’s disease and hypothyroidism. The finding of petrified ears may serve as a clinical sign and may warrant investigation of endocrine disorders.^3^
E. Patients are more likely to present with unilateral involvement – Incorrect. Bilateral involvement of the ears is more common.

## Conflicts of interest

None disclosed.
